# Deformity progression in congenital posteromedial bowing of the tibia: a report of 44 cases

**DOI:** 10.1186/s12891-020-03408-w

**Published:** 2020-07-03

**Authors:** Giovanni Luigi Di Gennaro, Giovanni Gallone, Edgar Alejandro Martinez Vazquez, Leonardo Marchesini Reggiani, Costantina Racano, Eleonora Olivotto, Stefano Stilli, Giovanni Trisolino

**Affiliations:** 1 Unit of Pediatric Orthopaedics and Traumatology, IRCCS Istituto Ortopedico Rizzoli, Bologna, Italy; 2 National Institute of Rehabilitation “Luis Guillermo Ibarra Ibarra”, Mexico City, Mexico; 3 RAMSES Laboratory, RIT Department, IRCCS Istituto Ortopedico Rizzoli, Bologna, Italy

## Abstract

**Background:**

congenital posteromedial bowing of tibia (CPMBT) is a very rare birth defect, characterized by shortened bowed leg and ankle deformity. We described a single institution experience in the management of CPMBT.

**Methods:**

we identified 44 CPMBT in 44 children. The age at presentation was 5.5 ± 5.6 years and the mean age at the final review was 10.1 ± 4.8 years. Radiographic evaluation included the antero-posterior and lateral inter-physeal angle (AP-IPA and L-IPA), the limb length discrepancy (LLD), the morphology of the distal tibia and the lateral distal tibial angle (LDTA). During the study period, 26 children underwent surgical treatment.

**Results:**

the estimated curves showed a progressive spontaneous correction of both AP-IPA and L-IPA during growth, but a progressive increase of the LLD. The L-IPA showed a more predictable behaviour while the AP-IPA showed a scattered correction, with a wider variation of the estimated final angle. The final LDTA was 85.3° ± 4.2° and was correlated with the L-IPA (*r* = 0.5; *p* = 0.02).

Among the 26 children who underwent surgical treatment, 23 cases had limb lengthening, 1 case had contralateral epiphysiodesis, 1 child underwent tibial osteotomy, 1 patient was treated by hemiepiphysiodesis of the distal tibia to correct ankle valgus deformity.

**Conclusions:**

our study described the largest case series of CPMBT. A combination of surgical treatments, in a staged surgical process, should be tailored to the developmental characteristics of this abnormality. An experience-based algorithm of treatment is also proposed. Further studies are needed to understand which is the best strategy to correct this deformity during childhood.

**Level of evidence:**

level IV prognostic study.

## Background

Congenital posteromedial bowing of the tibia (CPMBT) is a very rare birth defect, firstly fully described in 1949 by Heyman and Herndon [1]. It has been generally considered a benign, self-solving condition, in contrast to the anterolateral bowing, associated with congenital pseudarthrosis of the tibia, and the anteromedial bowing, associated with fibular hemimelia [2].

CPMBT is obvious at birth, with a notable shortening and bowing of the leg and the foot resting dorsiflexed against the tibial shaft [2–5]. Although the cause of CPMBT remains unknown, a potential role of amniotic strains has been hypothesized [6]. This condition is generally unilateral and not associated with other abnormalities [1, 7].

Several authors demonstrated spontaneous improvement of the tibial bowing within the first 3–4 years of life. Conversely, the limb length discrepancy (LLD) increases with age, until it reaches 4–7 cm at skeletal maturity [2, 3]. Studies have shown that the amount of the tibial bowing at birth is positively correlated to the LLD at maturity [2, 3, 8]. Moreover, some authors noticed residual ankle valgus at maturity, but its incidence and relationship with the leg deformity have not been established [2]. To date, several therapeutic options have been proposed for the management of CPMBT, but the treatment of choice remains controversial.

Therefore, we investigated a series of children presenting with CPMBT, treated at a single institution. We aimed to explain the behaviour of CPMBT during growth and the relationship between the tibial bowing, the leg shortening and the ankle deformity. These aspects could be useful in order to suggest a possible rationale of treatment.

## Methods

### Case series

The present study is a retrospective analysis of medical charts and radiographs of children affected by CPMBT, who were admitted at the Department of Pediatric Orthopedics and Traumatology from 1972 to 2016. Our institution is a tertiary referral center for pediatric orthopedics and traumatology, highly specialized in the treatment of complex deformities of the lower limb. All the charts and radiographs were analyzed by independent observers, who were not involved in the decision process about treatment and surgical management of the patients.

During the study period, 44 CPMBT were identified in 44 children, 27 boys and 17 girls. All children had unilateral involvement and no cases were excluded from the present study. The right side was affected in 25 children whereas the left side was affected in 19. The age at presentation was 5.5 ± 5.6 years (range 0–15) and the mean age at the final review was 10.1 ± 4.8 (range 0–16). Twenty-six patients underwent surgery during the study period, while the follow-up is still ongoing and surgery not yet planned for the remaining eighteen patients. Overall, Twenty-five children reached skeletal maturity at the time of final review; of them, twenty-four received definitive surgical treatment and one boy is waiting for tibial lengthening.

### Radiographic evaluation

**Fig. 1:**
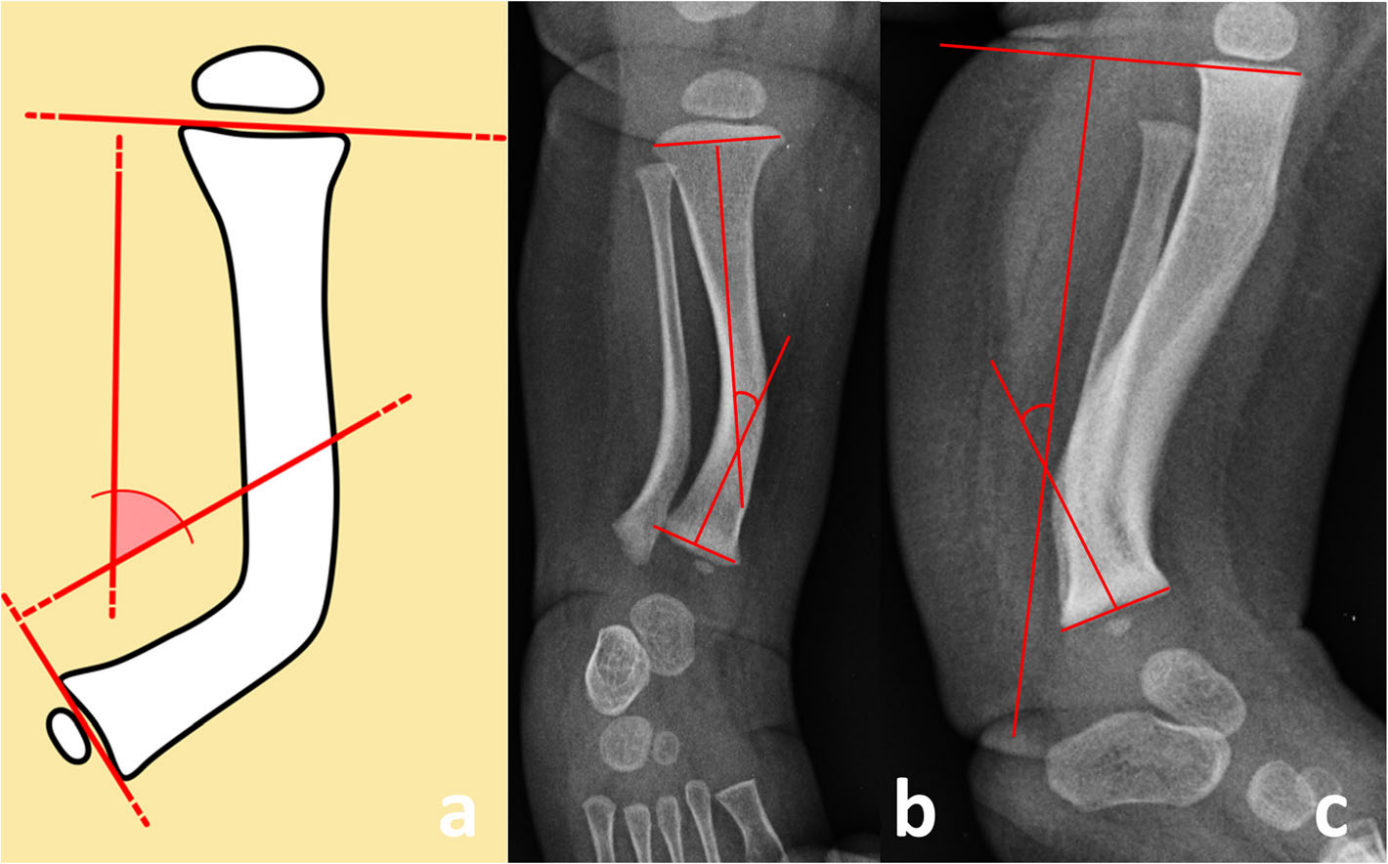
Schematic drawing of the tibia (1**a**), anteroposterior (1**b**) and lateral (1**c**) radiographs of the leg in a 1-month old baby. The interphyseal angle (IPA) is measured between a line perpendicular to the proximal physis and a line perpendicular to the distal physis on a true anteroposterior (AP-IPA) and a true lateral (L-IPA) view of the leg, respectively

**Fig. 2:**
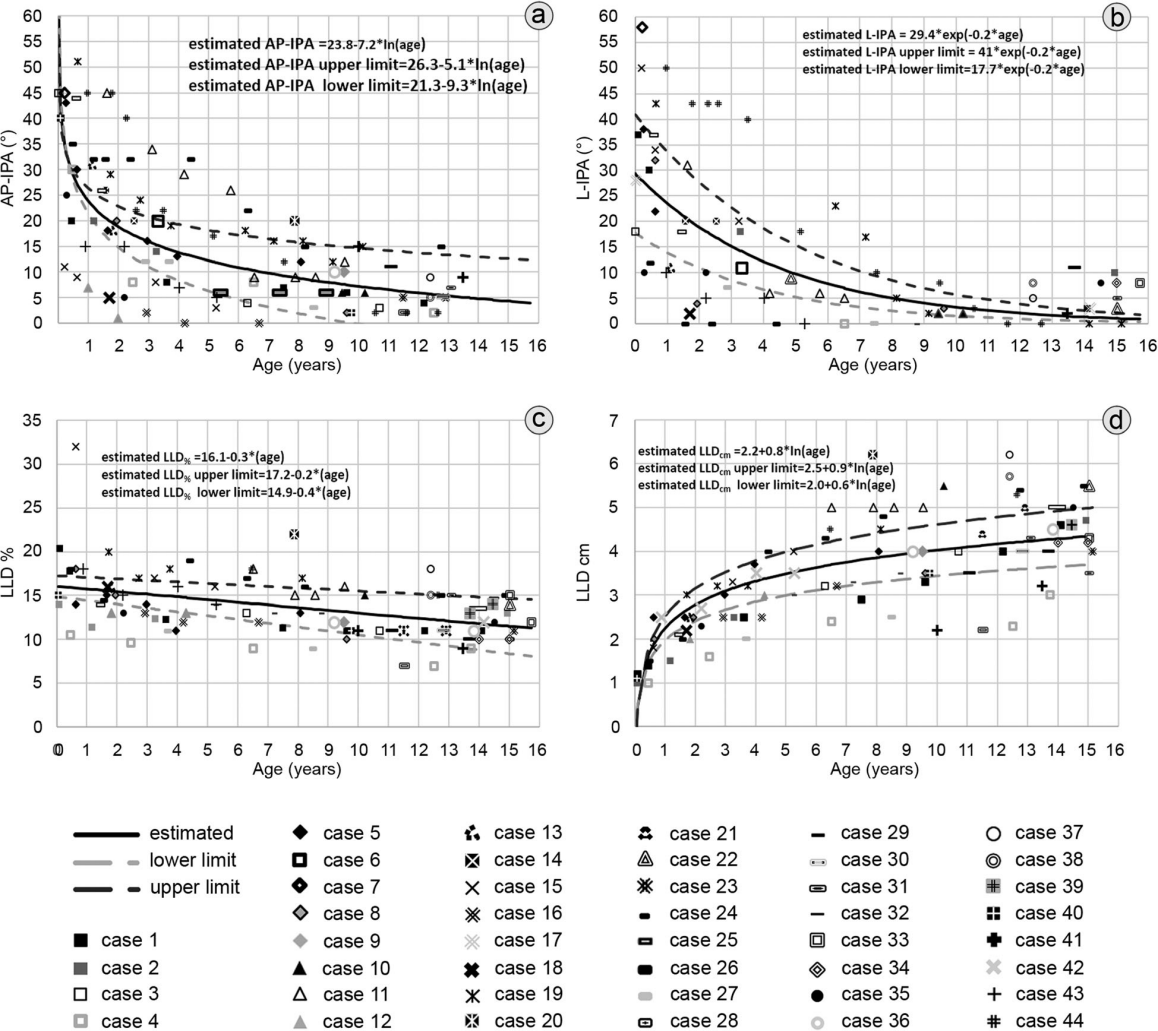
graph illustrating the estimated (black straight lines) spontaneous variation over time and the 95% confidence interval upper (dark grey dashed lines) and lower (light grey dashed lines) limits of the anteroposterior interphyseal angle (AP-IPA: Fig. 2**a**), the lateral interphyseal angle (L-IPA: Fig. 2**b**), the limb length discrepancy expressed as percentage shortening, as compared with the opposite side (LLD%: Fig. 2**c**), and as crude length (LLDcm: Fig. 2**d**)

The following variables were measured on serial sequential radiographs in order to assess the initial deformity and the spontaneous remodeling:1) the anteroposterior interphyseal angle (AP-IPA) and the lateral interphyseal angle (L-IPA), that are the angles measured between a line perpendicular to the proximal physis and a line perpendicular to the distal physis, on true anteroposterior and lateral views of the leg, respectively [2] Positive AP-IPA indicates medial bowing, while negative AP-IPA indicates lateral bowing. Positive L-IPA indicates posterior bowing, while negative L-IPA indicates anterior bowing (Fig. 1);

2) the leg length discrepancy (LLD) measured on long standing radiographs. The difference was expressed as crude length (LLDcm) and as percentage shortening as compared with the opposite side (LLD%);

3) the level of the distal fibular growth plate was graded according to the method of Malhotra et al. [9] (Fig. 1S), while the extent of the wedging of the distal tibial epiphysis was graded according to the method of Shapiro et al. (Fig. 2S) [10].

4) the lateral distal tibial angle (LDTA), medial proximal tibial angle (MPTA), anatomical lateral distal femoral angle (aLDFA) hip-knee-ankle angle (HKA) and mechanical axis deviation (MAD) measured on long standing radiographs in children approaching skeletal maturity [11, 12].

### Statistical analysis

Data were entered in Excel, SPSS and nlme package in R. Continuous variables were expressed as mean ± standard deviation (SD), while dichotomous or ordinal variables were expressed as percentage and 95% confidence interval (CI). Exploratory univariable and multivariable analyses were performed to assess the relationships among the parameters of CPMBT.

Normality was tested using the χ2 test for categorical variables and the Kolmogorov-Smirnov test for continuous variables. The Spearman’s Rho correlation test was used to investigate the relationships among continuous variables. The differences between groups were determined using the Fisher exact test for categorical variables and the independent sample t-tests for continuous variables with normal distribution. Variables with skewed distributions (Kolmogorov–Smirnov test, *p* < 0.05) were tested with the Mann– Whitney U-test.

In order to predict the spontaneous progression of both the deformity and the length discrepancy among patients over time, generalized linear mixed models, fitted by the maximum likelihood, were performed. A separate model was implemented for each parameter using the subject “patient” as a random factor. The best fitting model was chosen using the ANOVA R function, that compares the AIC, BIC and logLik values. A *p*-value of < 0.05 was considered statistically significant, and all reported *p*-values were 2-sided.

## Results

The estimated curves showed a progressive spontaneous correction of both the AP-IPA and the L-IPA (Fig. 2a-b and Table 1), and a slight decrease in LLD% (Fig. 2c), but a progressive increase of the LLDcm (Fig. 2d), until a final estimated discrepancy of 4.3 cm (95% CI 3.7–5.0).

Concerning the predictive model of spontaneous correction, we found that the best fitting curve was log-linear for the AP-IPA and LLDcm, exponential for the L-IPA and linear for the LLD%. Yet, the L-IPA showed a more predictable behaviour while the AP-IPA showed a scattered correction, with a wider variation of the estimated final angle (Table 1).

**Table 1:** estimated mean and 95% confidence interval (CI) of antero-posterior interphyseal angle (AP-IPA) and lateral interphyseal angle (L-IPA) by age, according to the predictive model of spontaneous correction

AGE (years)	AP-IPA (°)		L-IPA (°)	
	ESTIMATED MEAN	95% CI	ESTIMATED MEAN	95% CI
0	40	38–43	29	17–40
1	24	21–26	24	14–34
2	19	15–23	20	12–27
3	16	11–21	16	10–23
4	14	8–19	13	8–18
5	12	6–18	11	7–15
6	11	5–17	9	5–12
7	10	3–16	7	4–10
8	9	2–16	6	4–8
9	8	1–15	5	3–7
10	7	0–15	4	2–6
11	6.5	-1 – 14	3	2–5
12	6	−2 – 14	3	2–4
13	5	−3 – 13	2	1–3
14	5	−3 – 13	2	1–2
15	4	−4 – 12	1	1–2
16	4	−4 – 12	1	1–2
17	3	−5 – 12	1	1–1
18	3	−6 – 12	1	0–1

**Fig. 3:**
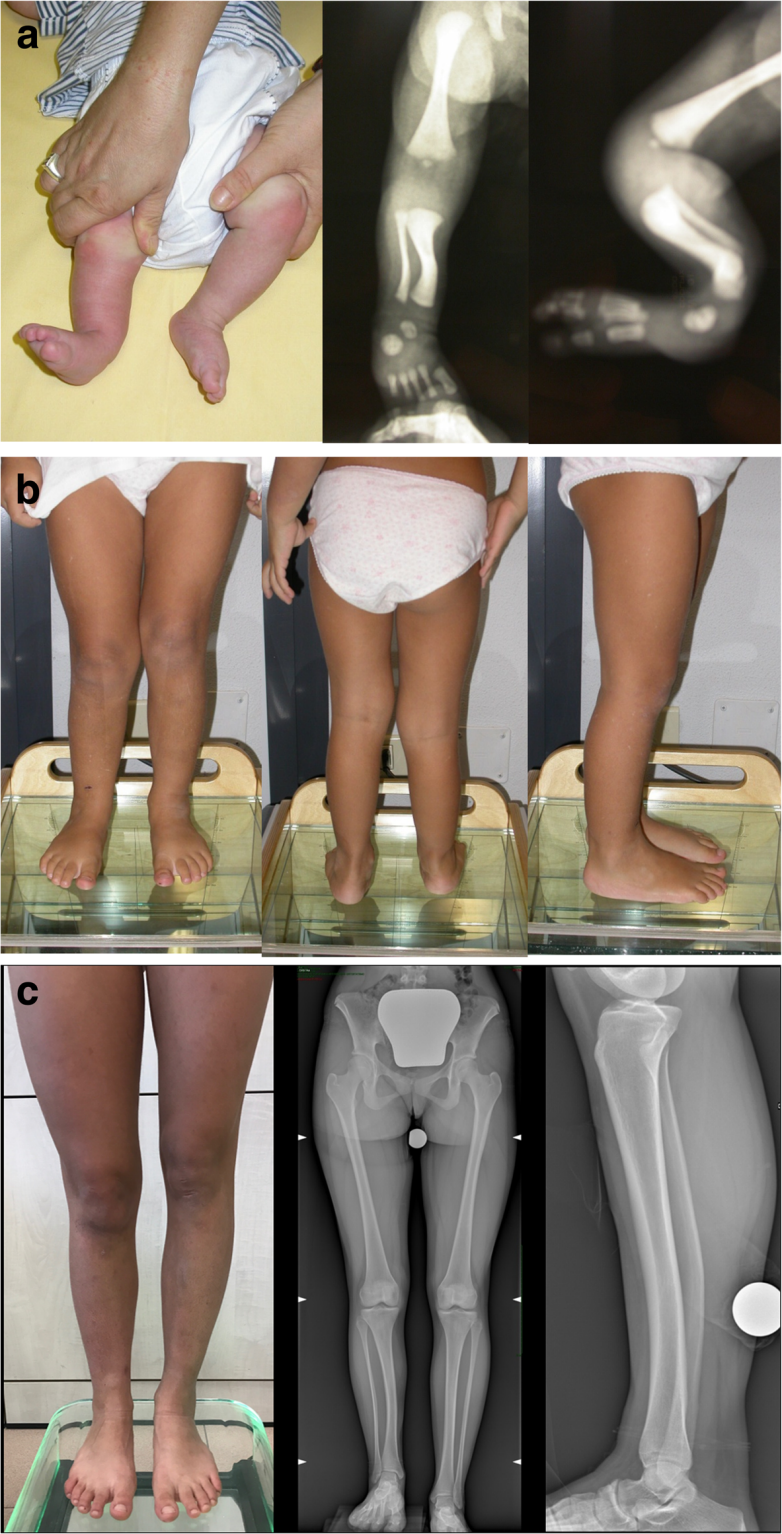
clinical and radiographic appearance of a child presenting with CPMBT, demonstrating the spontaneous progression of the deformity. Figure 3**a**: at 6 months of age; 3**b**: at 3.5 years of age; 3**c** at 15 years of age

We found almost perfect correlation between AP-IPA and L-IPA (eta-square = 0.81; *p* < 0.0005), and substantial correlation between AP-IPA and LLD% (eta-square = 0.69; *p* < 0.0005). the LDTA, measured at the latest follow-up, was 85.3° ± 4.2° (range 72° - 90°) and was correlated with the L-IPA (*r* = 0.5; *p* = 0.02). With the cases available, we did not find any relationship between the LDTA and Malothra score, Shapiro score MPTA, aLDFA, HKA, MAD. An illustrative case is showed in Fig. 3.

Twenty-six patients (mean age: 12.8 ± 2.7 years; range 5.3–15.7) underwent surgery during the study period.

The first case of CPMBT was diagnosed in our hospital in 1972 and was treated in 1978, at the age of 6 years. The patient sustained tibial lengthening by unilateral Wagner external fixator, then he underwent plate fixation after 2 months. The patient further developed delayed union treated with autologous bone graft and a subsequent fracture of the regenerated bone, treated by intra-medullary nailing.

Two patients had intermediate surgical treatment during the study period.

**Fig. 4:**
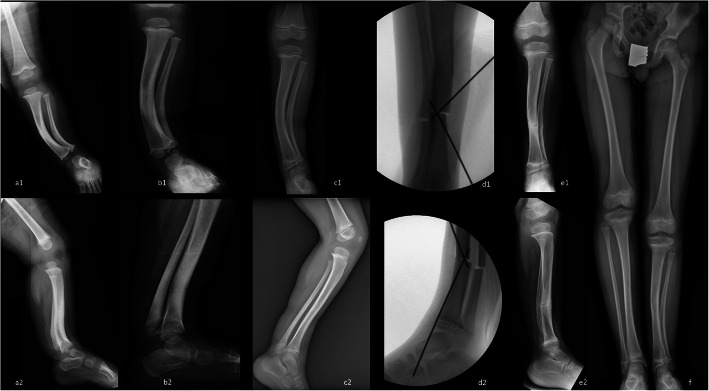
illustration showing the radiographs of a child affected by CPMBT, undergoing tibial osteotomy. 4**a**1–2: anteroposterior and lateral views at 19 months of age. 4**b**1–2: anteroposterior and lateral view at 3.5 years of age. 4**c**1–2: pre-operative anteroposterior and lateral radiographs at 5.8 years of age, showing a residual AP-IPA of 26°. 4**d**1–2: intra-operative anteroposterior and lateral views. 4**e**1–2: post-operative anteroposterior and lateral radiographs 4 months after surgery showing complete bone healing. 4**f**: long standing radiographs at 11.5 years of age. The patient had a residual deformity with an AP-IPA of 9° a L-IPA of 5° a residual LLD of 5.5 cm (16%) and a LDTA of 70°

In one patient, a severe tibial deformity persisted at the age of 5.8 years, with an AP-IPA of 26°, a L-IPA of 6° and a LLD 5 cm (18%) (Fig. 4a-c). The patient was treated by corrective tibial osteotomy at the apex of the deformity, stabilized with two Kirschner wires (Fig. 4d). At the latest follow up, 3.5 years after surgery, the patient had a residual deformity with an AP-IPA of 9° a L-IPA of 5° a residual LLD of 5 cm (16%) and an ankle valgus deformity of 70° (Fig. 4e). To date, the patient is waiting for final correction and leg lengthening.

**Fig. 5:**
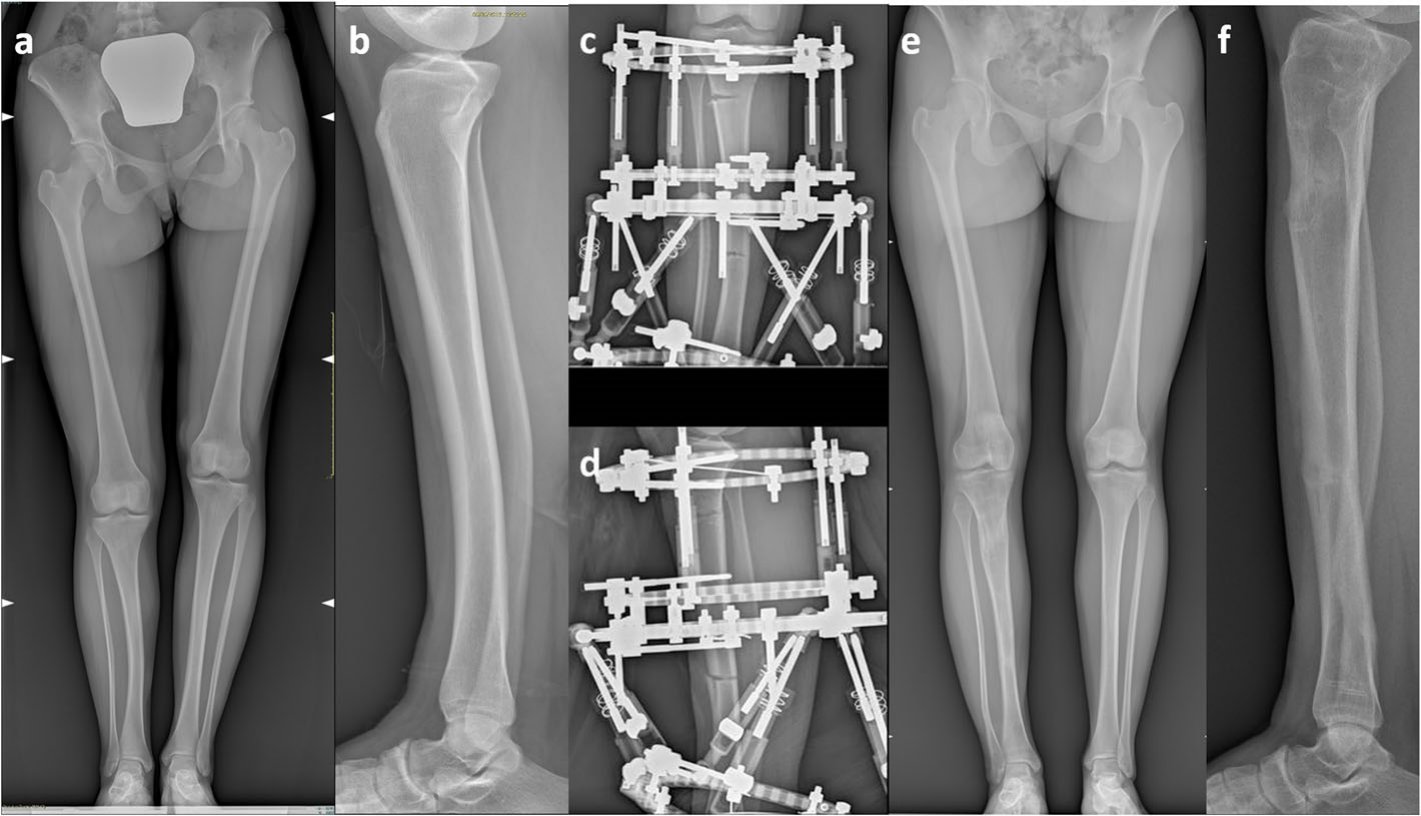
illustration showing the radiographs of a 14-years girl affected by CPMBT undergoing bifocal lengthening by circular hexapod external fixation device (TL-HEX Truelok Hexapod System® Orthofix srl - Verona – Italy) to equalize a LLD of 5 cm with a residual antero-posterior bowing of 8°. The deformity correction was achieved at the distal osteotomy with further lengthening at the proximal osteotomy.5**a**-**b**: pre-operative long-standing and lateral radiographs. 5**c**-**d**: early post-operative radiographs (antero-posterior and lateral view). 5**e**-**f**: postoperative long standing and lateral radiographs at the final follow-up one year after the external fixator removal

**Fig. 6:**
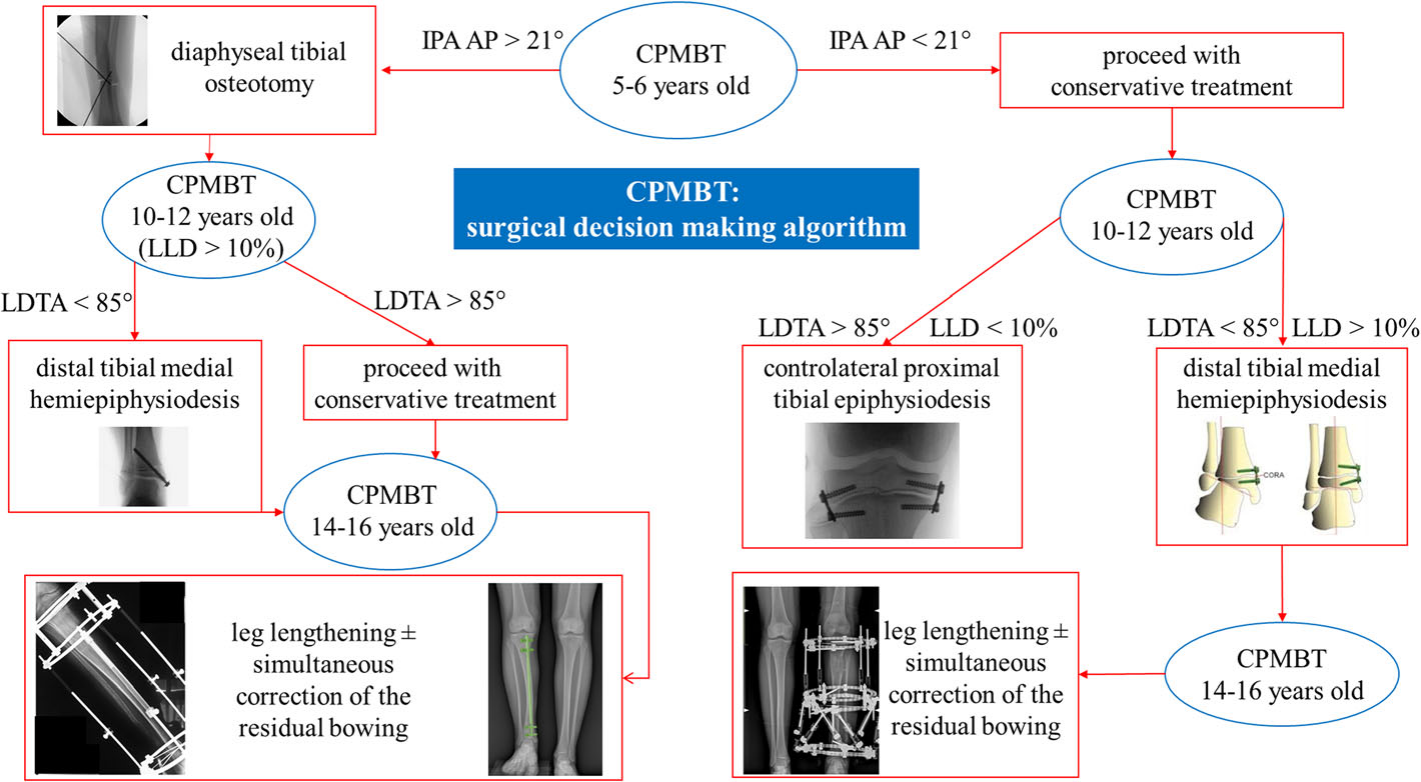
Experience based algorithm of treatment in CPMBT

One patient underwent temporary medial hemiepiphysiodesis of the distal tibia at the age of 10.4 years, for correcting ankle valgus (LDTA = 79°). At the latest follow-up visit, 2 years after the operation, the ankle axis was restored (LDTA = 90°) and the patient is currently waiting for final lengthening, to equalize a residual LLD of 4 cm (11%).

One patient, who presented with residual LLD of 2.2 cm (7%) and no relevant deformities of the tibial and ankle axis, was treated at the age of 11.4 years with contralateral epiphysiodesis of the proximal tibia. The patient achieved limb length equalization 30 months after surgery, without further complications.

Twenty-two patients were treated at skeletal maturity (13.7 ± 1.8 years). The pre-operative radiographic data are summarized in Table 2. All these patients received leg lengthening by circular external fixation. We never bridged the ankle, but preferred foot splints attached to the fixator by elastic bands, in order to promote immediate full weight bearing and active rehabilitation of the ankle. Bifocal lengthening was accomplished in one case (Fig. 5), in which the deformity was corrected through the distal osteotomy, while lengthening was carried out at the proximal osteotomy, in order to reduce the time in frame, due to the better healing potential of the metaphyseal osteotomy. In this group, the final limb equalization was achieved in all patients, but we experienced 11 complications in 8 patients. According to Lascombes et al. [13], there were 2 grade IIa complications (2 operations to change or modify the frame) and 9 grade IIIa complications (1 delayed bone healing, 3 fibular nonunion, 1 knee joint stiffness; 2 achilles tendon shortening; 2 malalignment with residual anterior bowing of the tibia). Moreover the final LDTA was 83.3° ± 5.2° (range 72° - 90°), with 9 patients out of 14 showing a residual LDTA < 85°.

**Table 2:** pre-operative radiographic characteristics of the 22 patients that underwent leg lengthening by Ilizarov external fixation. AP-IPA: Anterior-Posterior Inter-Physeal Angle. L-IPA: Lateral Inter-Physeal Angle. LLD: leg length discrepancy calculated in absolute (cm) and relative (%) terms, compared to the contralateral side. MPTA: Medial Proximal Tibial angle. aLDFA: anterior Lateral Distal Femoral angle. HKA: Hip-Knee-Ankle angle. MAD: Mechanical Axis Deviation is defined as the distance from the midpoint of the tibial plateau to a line connecting the midpoints of the hip and ankle joint. In varus alignment the MAD was defined as a positive value in millimeters, while in cases of valgus position, the MAD was determined as a negative value

Radiographic parameter	Mean ± SD (range)
AP-IPA	6.8 ± 4.6 (0–20)
L-IPA	4.8 ± 3.8 (0–11)
LLD (%)	12.9 ± 3.0 (9–22)
LLD (cm)	4.6 ± 0.9 (3–6.2)
LDTA	83.3 ± 5.2 (72–90)
MPTA	82.8 ± 2.6 (80–88)
aLDFA	79.7 ± 2.3 (74–84)
HKA	4.8 ± 4.2 (0–18)
MAD (mm)	−5.2 ± 7.0 (3 – − 24.8)

## Discussion

This study reports the largest series of children with CPMBT in the current literature. To date, about 200 cases of CPMBT have been reported in the available literature [1–5, 7, 8, 14–18]. For years CPMBT has been considered a benign self-solving condition, due to the virtually absent risk of fracture or pseudarthrosis and the natural tendency to the spontaneous resolution, with minimal residual deformity. Nonetheless, we noticed that the spontaneous correction of the bowing was sometimes incomplete, the LLD was frequently wide and a residual ankle valgus could persist at the end of growth. We found some interesting correlation between the AP-IPA and the L-IPA, and between the AP-IPA and the LLD%. In other words, the greater is the angular deformity at birth, the wider will be the LLD at skeletal maturity. This finding is confirmed by previous reports [2, 3, 8, 18].

We found that the posterior bow corrected more efficiently than the medial bow, but the spontaneous correction of the posterior bow was accompanied by an increase of the ankle valgus. This phenomenon was also noticed by other authors [2, 3] and could be explained by a possible pathogenetic hypothesis of CPMBT, namely the “amniotic band theory” [6]. According to this theory, CPMBT is caused by a partial rupture of the amniotic sac, during pregnancy. This rupture can lead to a focal growth arrest of the antero-lateral distal portion of the leg, causing the typical clinical aspect of CPMBT. Then, after birth, the diaphyseal deformity of the leg improves spontaneously, according to the Wolff’s law, thus the leg corrects more efficiently in the sagittal plane (plane of walking) rather than in the coronal plane. It is plausible that part of the spontaneous diaphyseal improvement is determined by an asymmetric growth of the distal tibial physis, leading to consequent ankle valgus [2, 9, 10]. However, our study cannot definitely confirm this hypothesis, since we did not find significant correlations among Malothra, Shapiro scores and LDTA; further studies are needed to clarify this effect.

The management of CPMBT is still debated since there is no complete evidence about the optimal treatment strategy. Given the tendency to the spontaneous correction of the deformity, some authors suggest conservative treatment consisting of manipulation, serial casting, orthoses and shoe lifts; then, a limb equalization is proposed during late childhood, if needed [4, 7, 14]. Currently, there is no evidence that the use of braces and orthoses may improve the angular correction in CPMBT, since it occurs spontaneously and independently by the use of orthoses. The main goal of braces and orthoses is to aid walking and balance, while the patient is too young for the surgical treatment.

There are three reasons for surgical intervention in CPMBT: equalize the LLD, correct the ankle valgus and correct the residual bowing of the tibia [2]. Nonetheless, there are no clearly defined guidelines for the surgical treatment of CPMBT.

Regarding the LLD, we found that the final discrepancy at skeletal maturity averaged 4.3 cm, corresponding to 13% of the length of the unaffected leg; this ratio, remains rather constant during growth, as confirmed by many previous reports [2–5, 18]. In our series, all the children who reached skeletal maturity underwent limb lengthening by circular external fixator while only one child underwent contralateral epiphysiodesis. Albeit contralateral epiphysiodesis has been recommended in CPMBT, due to the lower risk of complications compared to limb lengthening [3], aesthetical issues can raise due to the loss of body height. Moreover, recent concern has mounted regarding the potential risk of compromising the morphology of the proximal tibia, when a large, congenital LLD must be addressed [19, 20]. Therefore, we suggest to reserve this treatment only for children in which the LLD% at 10–12 years is less than 10%, corresponding about 2 to 3 cm. Regarding the tibial bowing, the majority of cases improved spontaneously by the end of growth. Our behaviour consisted in a “waiting strategy”, using braces and orthoses until skeletal maturity, then, correcting in a single stage the length and the potential residual bowing. In our opinion, this strategy should reduce the risks for the patient and the costs for the health service. Nonetheless, a more pronounced reduction of the angular deformity was noticed during the first 6 years of life; thereafter, the rate of spontaneous correction decreased markedly. Many authors reported that, in CPMBT, the greatest rate of correction is observed during the first year of life, then, rapidly decreases until the age of four [2–4, 15, 18]. This aspect may have practical implications, because an extreme bowing of tibia in a school-age child might hamper even the possibility of using braces to aid walking. In this scenario, some authors suggested early corrective osteotomy at the apex of the deformity, by the age of 3 to 6 years [2–4, 15, 16]. It has been argued that the intense periosteal activity at this age allows for early bone healing of the diaphyseal osteotomy; furthermore, the overgrowth of the tibia due to the physeal stimulation and the tibial straightening could potentially contribute to the leg length equalization [15]. We treated only one case by early corrective osteotomy of the bowed tibia: although we achieved a rapid healing of the osteotomy and a perfect alignment of the tibia, we observed a progressive partial relapse of the bowing, an important ankle valgus, while the LLD remained unchanged. These finding are consistent with those reported by Johari et al. [4], suggesting that the early tibial osteotomy should be proposed only in case of severe, disabling bowing, as an intermediate treatment, to avoid complex bracing and allow walking with simple foot orthosis or shoe lift. Based on our experience, we recommend early tibial osteotomy, if the AP-IPA does not decrease below 21° by the age of 6 years (corresponding to more than two standard deviations of AP-IPA within our cohort). Nonetheless, further studies are needed to confirm this threshold.

Another reason to recommend early tibial osteotomy, is related to the possibility to perform intramedullary lengthening by telescopic nails at skeletal maturity [21]. This technique has been reported as safe, effective and more tolerated by the patients, in comparison with external fixation. Nevertheless, intramedullary nailing is more simple, safer and more effective when applied to a straight tibia rather than to a bowed tibia. Yet, simultaneous correction of the bowing and lengthening by circular external fixation is not simple, requires high compliance by the child and the parents, high proficiency with the technique and it has high risk of complications. Although we have about 40-year experience with the Ilizarov technique [22], we experienced a relevant rate of moderate and severe complications, in line with other reports: Kaufman et al. [5] reported 17 mild to severe complications in 11 CPMBT treated by external fixation; Johari et al. [4] described complications in all the 6 cases treated by external fixation; Wright et al. [18] reported 16 complications in 17 children treated by external fixation. Probably, computer-assisted hexapods devices could be more effective in achieving lengthening and correction compared to the conventional Ilizarov method, reducing the lengthening index for faster correction, but potential advantages must be balanced by costs, since the Ilizarov device is much less expensive, compared to Hexapod [23]. Furthermore, we suggest to perform the lengthening procedures closer to the skeletal maturity, since we did not experience any recurrence of the limb length inequality, in contrast with other authors, who reported relapse of the limb length inequality, if the lengthening procedure was performed during growth [2–5, 18].

Finally, in our series about one third of children with CPMBT presented a valgus ankle by the end of growth (LDTA < 85°). This issue has been reported previously [2, 4]. Although the normal range of the LDTA has been established [11, 12], an exact cut-off to define a pathologic deformity has not been clearly defined, with proposed values varying from 5° to 10° of valgus [20, 21, 24, 25]. In our series, only one patient underwent distal tibial hemiepiphysiodesis to treat ankle valgus. Nonetheless, it is our opinion that a medial distal tibial hemiepiphysiodesis should be performed if the valgus inclination of the distal tibial articular surface persists by the age of 10 to 11 years, when sufficient growth potential is still present [18, 19]. This simple procedure can effectively realign the ankle by minimally invasive surgery, reducing complications and avoiding more demanding surgeries at the end of growth [24–30]. The residual “S” shape within the long axis of the tibia could not require further correction, if the joint lines of knee and ankle are parallel to the floor.

Based on our findings and on the available literature, we identified crucial steps and thresholds for an experience-based algorithm of surgical decision-making (Fig. 6): 1) diaphyseal tibial osteotomy could be performed at 5–6 years of age, in case of severely disabling bowing deformity (generally AP-IPA > 21°);

2) distal tibial medial hemiepiphysiodesis could be indicated at 10–11 years, in case of important ankle valgus (LDTA < 85°);

3) minor LLD (discrepancy ≤10%, corresponding to about 3 cm) can be addressed by contralateral epiphysiodesis at the age of 11–12 years;

4) severe LLD (discrepancy > 10%) should be treated close to skeletal maturity (13–15 years) by leg lengthening, possibly combined with simultaneous correction of the residual bowing.

### Limitations

Despite our study describes the largest series of CPMBT in the available literature, some limitations must be highlighted. We conducted a retrospective analysis of cases collected across more than 40 years, sometimes with incomplete information and missing data. Twelve cases out of forty-four had no sufficient radiographic follow-up available for assessing the progression of the deformity. Furthermore, the cases were not collected uniformly at birth and 16 children had the first radiographic evaluation when they were older than 5 years. The majority of long leg radiographs were taken without the patient balanced on blocks. Although this unbalanced position should not significantly influence the assessment of the leg length discrepancy, it could introduce a source of bias in assessing the mechanical axis of the lower limb. Finally, almost half of cases did not reach skeletal maturity, thus not undergoing the final correction. We aim to maintain a close follow-up of those patients who have not received the definitive treatment and update our results within five to 10 years.

These limitations were encountered in all previous reports about CPMBT [2–5, 18], emphasizing the difficulty in obtaining complete information about rare abnormalities, where less than one case is diagnosed per year, even in highly specialized institutions. In order to address this issues and minimize biases, we used mixed effect models, a complex statistical approach that allows to maximize the prediction power from small and heterogeneous groups of subjects with missing data; nonetheless, we are aware that our results could be weakened by poor statistical power and precision.

## Conclusions

Despite its supposed benignity, CPMBT is a complex deformity, in which the spontaneous correction of the angular deformity is inconstant and incomplete, the LLD is generally wide, and a substantial ankle valgus can be observed by the end of growth. A combination of surgical treatments (osteotomies, epiphysiodesis or hemiepiphysiodesis, leg lengthening) in a staged multistep surgical process, should be tailored on the developmental characteristics of the deformity. This approach may accomplish the correction of the deformity, minimizing complications and failure, and helping surgeons and parents in the decision process. Further studies are needed to understand which is the best strategy to address this rare deformity during childhood.

## Supplementary information

**Additional file 1.** Fig. 1S: Illustration showing Malhotra’s grading system. The degree of ankle valgus is determined based on the level of the fibular growth plate. Grade 0 (normal): the fibular growth plate is at the level of the tibial plafond. Grade 1 (mild): the fibular growth plate is above the level of the tibial plafond but below the level of the distal tibial growth plate. Grade 2 (moderate): the fibular growth plate is at the level of the distal tibial growth plate. Grade 3 (severe): the fibular growth plate is above the level of the distal tibial growth plate.

**Additional file 2.** Fig. 2S: Illustration showing Shapiro’s grading system of wedging of the distal tibial epiphysis. Grade 0: No wedging of the distal tibial epiphysis is detectable. Grade 1: the wedging occurred from the central portion of the distal tibial epiphyseal surface and angled upwards and laterally but the lateral margin of the epiphysis remained well separated from the growth plate. Grade 2: the distal tibial epiphyseal surface sloped into the lateral margin of the growth plate. Grade 3: the distal tibial epiphyseal surface slanted into the growth plate in its lateral third rather than its lateral edge

## Data Availability

The datasets used and/or analyzed during the current study are available from the corresponding author on reasonable request.

## Abbreviations

CPMBT: Congenital posteromedial bowing of tibia
AP-IPA: Anteroposterior inter-physeal angle
L-IPA: Lateral inter-physeal angle
LLD: Limb length discrepancy
LDTA: Lateral distal tibial angle
SD: Standard deviation
95% CI: 95% confidence interval
ANOVA: Analysis of variance
AIC: Akaike information criterion
BIC: Bayesian information criterion
