# Supplementary figures and images for: Deformity progression in congenital posteromedial bowing of the tibia: a report of 44 cases
Source: BMC Musculoskelet Disord. 2020 Jul 3;21:430. doi: 10.1186/s12891-020-03408-w (PMC7334844; doi:10.1186/s12891-020-03408-w)

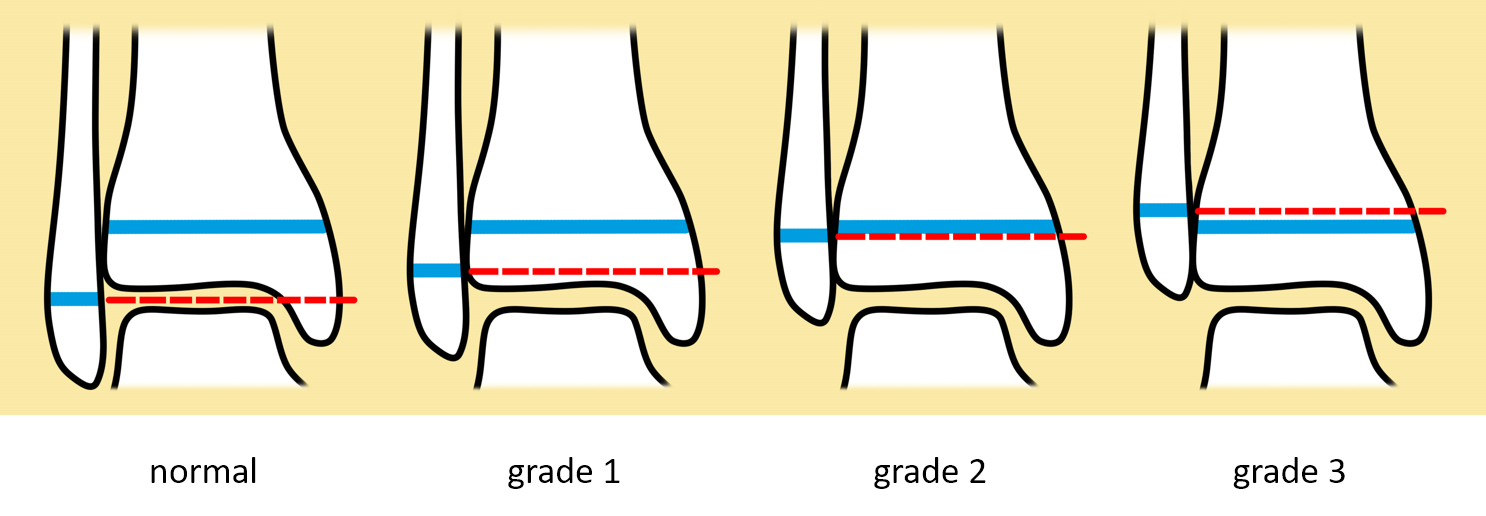

Supplement: Supplementary file 1 — Additional file 1. Fig. 1S: Illustration showing Malhotra’s grading system. The degree of ankle valgus is determined based on the level of the fibular growth plate. Grade 0 (normal): the fibular growth plate is at the level of the tibial plafond. Grade 1 (mild): the fibular growth plate is above the level of the tibial plafond but below the level of the distal tibial growth plate. Grade 2 (moderate): the fibular growth plate is at the level of the distal tibial growth plate. Grade 3 (severe): the fibular growth plate is above the level of the distal tibial growth plate. [file 12891_2020_3408_MOESM1_ESM.tif]

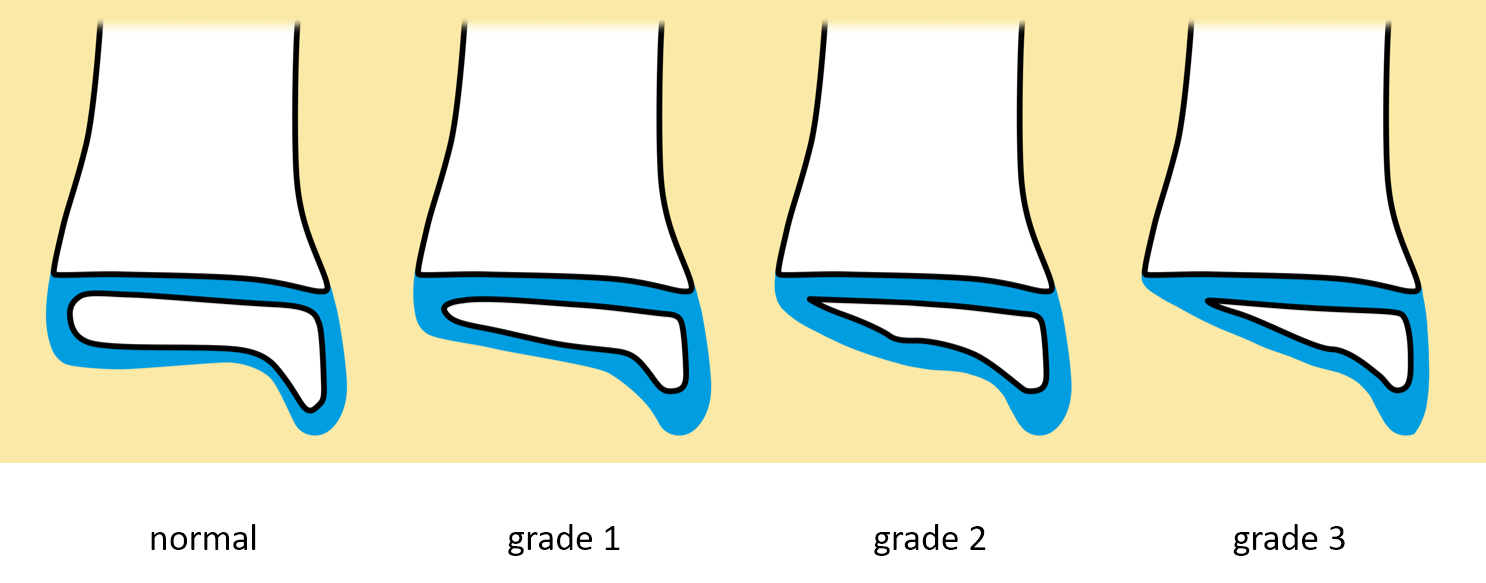

Supplement: Supplementary file 2 — Additional file 2. Fig. 2S: Illustration showing Shapiro’s grading system of wedging of the distal tibial epiphysis. Grade 0: No wedging of the distal tibial epiphysis is detectable. Grade 1: the wedging occurred from the central portion of the distal tibial epiphyseal surface and angled upwards and laterally but the lateral margin of the epiphysis remained well separated from the growth plate. Grade 2: the distal tibial epiphyseal surface sloped into the lateral margin of the growth plate. Grade 3: the distal tibial epiphyseal surface slanted into the growth plate in its lateral third rather than its lateral edge [file 12891_2020_3408_MOESM2_ESM.tif]
